# VETS AND VEGGIES: FEASIBILITY OF OFFERING A FREE ONSITE FARMER’S MARKET TO OLDER VETERANS

**DOI:** 10.1093/geroni/igae098.2836

**Published:** 2024-12-31

**Authors:** Elizabeth Dennis, Jake Drumheller, Kelly Barton-Ort, Jamie Giffuni, Jeffrey Beans, Odessa Addison

**Affiliations:** University of Maryland School of Medicine, Baltimore, Maryland, United States; Baltimore VA, Baltimore, Maryland, United States; Baltimore VA Medical Center, Baltimore, Maryland, United States; Baltimore and Loch Raven VAMC, Baltimore, Maryland, United States; Baltimore VA, Baltimore, Maryland, United States; University of Maryland School of Medicine, Baltimore, Maryland, United States

## Abstract

This project evaluated a 6-month, free, onsite farmer’s market offered through Gerofit, a health promotion program for older Veterans. Each week, 4-8 fresh produce items were delivered to the Gerofit gym facility as a strategy to overcome barriers related to fresh produce access. Participants self-selected items, taking as many or few items as preferred. Gerofit staff sent weekly emails with suggested recipes for produce preparation and recorded weekly leftover produce. An end of season administered questionnaire assessed Veterans’ perceived impact on diet, reasons for choosing weekly produce items and open-ended feedback. The most common weekly leftover items were leafy greens (e.g., lettuce, chard) and squash. Of 25 participants who completed questionnaires, 60% reported eating new types of produce because of the farmer’s market. Eleven participants reported trying a new item specifically because of Gerofit staff recommendations. When asked why they didn’t choose specific items, 15 reported being unsure of preparation methods and 10 reported disliking the item. When asked what participants did with the produce received, most (84%) said they cooked it using their own recipe. Nineteen participants (76%) reported that the farmer’s market saved them money, 72% reported that it made it easier for them to eat healthier, and 60% said it helped them eat a wider variety of produce. Veterans rated the weekly produce 9.7/10 (1-10 scale; 10 being most helpful). Future studies are needed to implement and evaluate strategies designed to increase overall participation, encourage trying new produce, and reduce weekly food waste.

